# Responses of macroalgae to CO_2_ enrichment cannot be inferred solely from their inorganic carbon uptake strategy

**DOI:** 10.1002/ece3.4679

**Published:** 2018-12-14

**Authors:** Luna M. van der Loos, Matthias Schmid, Pablo P. Leal, Christina M. McGraw, Damon Britton, Andrew T. Revill, Patti Virtue, Peter D. Nichols, Catriona L. Hurd

**Affiliations:** ^1^ Institute for Marine and Antarctic Studies University of Tasmania Hobart Tasmania Australia; ^2^ Marine Ecology University of Groningen Groningen The Netherlands; ^3^ Instituto de Fomento Pesquero (IFOP) Puerto Montt Chile; ^4^ Department of Chemistry, NIWA/University of Otago Research Centre for Oceanography University of Otago Dunedin New Zealand; ^5^ CSIRO Oceans and Atmosphere Hobart Tasmania Australia; ^6^ Antarctic Climate and Ecosystems Cooperative Research Centre Hobart Tasmania Australia

**Keywords:** carbon uptake strategy, carbon dioxide‐concentrating mechanism, CCM, CO_2_ enrichment, macroalgae, non‐CCM, ocean acidification, physiology

## Abstract

Increased plant biomass is observed in terrestrial systems due to rising levels of atmospheric CO_2_, but responses of marine macroalgae to CO_2_ enrichment are unclear. The 200% increase in CO_2_ by 2100 is predicted to enhance the productivity of fleshy macroalgae that acquire inorganic carbon solely as CO_2_ (non‐carbon dioxide‐concentrating mechanism [CCM] species—i.e., species without a carbon dioxide‐concentrating mechanism), whereas those that additionally uptake bicarbonate (CCM species) are predicted to respond neutrally or positively depending on their affinity for bicarbonate. Previous studies, however, show that fleshy macroalgae exhibit a broad variety of responses to CO_2_ enrichment and the underlying mechanisms are largely unknown. This physiological study compared the responses of a CCM species (*Lomentaria australis*) with a non‐CCM species (*Craspedocarpus ramentaceus*) to CO_2_ enrichment with regards to growth, net photosynthesis, and biochemistry. Contrary to expectations, there was no enrichment effect for the non‐CCM species, whereas the CCM species had a twofold greater growth rate, likely driven by a downregulation of the energetically costly CCM(s). This saved energy was invested into new growth rather than storage lipids and fatty acids. In addition, we conducted a comprehensive literature synthesis to examine the extent to which the growth and photosynthetic responses of fleshy macroalgae to elevated CO_2_ are related to their carbon acquisition strategies. Findings highlight that the responses of macroalgae to CO_2_ enrichment cannot be inferred solely from their carbon uptake strategy, and targeted physiological experiments on a wider range of species are needed to better predict responses of macroalgae to future oceanic change.

## 1 INTRODUCTION

Since the beginning of the industrial era, atmospheric CO_2_ concentrations have increased by 40% (IPCC, 2013). In terrestrial systems, there is evidence of a “CO_2_ enrichment” effect that is resulting in a higher plant biomass (Zhu et al., 2016) as the availability of CO_2_ for carbon fixation in photosynthetic organisms via the enzyme Rubisco increases. In coastal marine systems, macroalgae fulfill the same functional role as terrestrial plants; they are foundation species that provide food, habitat, and shelter to higher trophic levels (e.g., shellfish and fish), as well as a range of ecosystem services including nutrient cycling. In addition, they have a potential role in the blue carbon economy and climate mitigation (Bennett et al., 2015; Chung, Beardall, Mehta, Sahoo, & Stojkovic, 2011; Tuya, Wernberg, & Thomsen, 2008). By the end of the century, CO_2_ concentrations in seawater will increase by 200% under the RCP (*Representative Concentration Pathway*) 6.0 scenario (IPCC, 2013) and, like terrestrial plants, macroalgal productivity is predicted to increase (Koch, Bowes, Ross, & Zhang, 2013; Sunday et al., 2016).

The absorption of CO_2_ by seawater and the subsequent chemical reactions cause a suite of changes to the seawater carbonate system, termed ocean acidification: By 2100, the bicarbonate ion will additionally increase by 14% with a concurrent decline in pH of 0.3 units (RCP6.0 scenario; Hurd, Hepburn, Currie, Raven, & Hunter, 2009; IPCC, 2013; The Royal Society, 2005). In addition to taking up CO_2_ via diffusion, ~65% of macroalgae can utilize the bicarbonate ion directly from seawater (Kübler & Dudgeon, 2015). Bicarbonate use is an energy‐consuming process, and the derivation of CO_2_ from bicarbonate (for subsequent carbon fixation) is termed a carbon dioxide‐concentrating mechanism (CCM). Species that solely rely on the diffusive uptake of CO_2_, termed non‐CCM species (mostly Phylum Rhodophyta), were thought to be rare (Kübler & Dudgeon, 2015; Raven, Ball, Beardall, Giordano, & Maberly, 2005). However, Cornwall, Revill, and Hurd (2015) discovered that up to 90% of some populations were non‐CCM species in subtidal reef habitats in Tasmania, southern Australia, and on average 35% of macroalgae globally are now considered non‐CCM (Kübler & Dudgeon, 2015).

Extensive field surveys have been conducted to study CCM and non‐CCM species in temperate (Cornwall et al., 2015; Hepburn et al., 2011) and tropical systems (Diaz‐Pulido, Cornwall, Gartrell, Hurd, & Tran, 2016), and along a gradient of CO_2_/pH in a volcanic vent in Italy (Cornwall et al., 2017). These surveys indicate that the responses of macroalgae to CO_2_ enrichment will depend on their mechanism(s) of inorganic carbon uptake, that is, their carbon uptake strategies (Hepburn et al., 2011). For CCM species, the predominance of the bicarbonate ion in seawater (90% under current pH) may mean that their growth is not limited by dissolved inorganic carbon (DIC) supply and there will be no future CO_2_ enrichment effect. However, field surveys have illustrated that within CCM species we can further distinguish between (a) those that are able to use the 200% additional CO_2_ predicted for the future and (b) those that cannot (Cornwall et al., 2017; Hepburn et al., 2011; Kübler & Dudgeon, 2015): (a) Species able to use additional CO_2_ either exhibit a low affinity for DIC (carbon‐limited CCM species) or have CCMs that can be downregulated (i.e., decreased CCM activity); (b) CCM species that are considered unresponsive to future CO_2_ concentrations have either a high affinity for CO_2_ or CCMs that cannot be downregulated under elevated CO_2_ conditions (Cornwall et al., 2017). In contrast, growth and photosynthesis of non‐CCM species are thought to be limited under current CO_2_ concentrations and they are predicted to benefit from the future 200% increase in dissolved CO_2_ (Cornwall et al., 2015; Hepburn et al., 2011; Kübler & Dudgeon, 2015).

Carbon uptake strategies in macroalgae can be determined by analyzing their carbon stable isotope composition (δ^13^C) (Maberly, Raven, & Johnston, 1992): CO_2_ is more depleted in ^13^C than HCO_3_^−^ and thus has a lower δ^13^C value. Macroalgae that possess a CCM typically have δ^13^C values ranging between −30‰ and −10‰, whereas a lack of a CCM is indicated by δ^13^C <−30‰. A change in the δ^13^C value of the macroalgal tissue indicates a change in carbon uptake strategy and can also indicate biochemical changes in the macroalgae such as the use of sugars and lipid synthesis (Raven et al., 2002). Macroalgae generally exhibit significant changes in both lipids and fatty acids with changing environmental conditions. Such biochemical changes can be observed in the biomass very shortly after exposure (≤7 days) as an acclimation mechanism in membrane and/or storage lipids (Al‐Hasan, Hantash, & Radwan, 1991; Gosch, Lawton, Paul, Nys, & Magnusson, 2015). Elevated CO_2_ concentrations have been shown to either increase or decrease the lipid production of several species of algae, especially when combined with nutrient limitation (Bermúdez et al., 2015; Sun, Chen, & Du, 2016). CO_2_ enrichment could therefore not only result in increased growth rates, but also lead to changes in lipid storage. Whether a species will invest in growth or lipid storage may depend on their carbon uptake strategy. Species that are currently saturated for DIC (i.e., CCM species) may use the elevated DIC to invest in lipid storage, since their growth rates are not limited by carbon. Species whose growth rates are currently limited by DIC (i.e., non‐CCM species) may increase their growth rates first before investing in storage compounds.

Given that both CO_2_ and bicarbonate concentrations are increasing in seawater, and macroalgae have a variety of inorganic carbon uptake strategies (Diaz‐Pulido et al., 2016), determining the physiological and growth responses of macroalgae to future CO_2_ enrichment is complex. Despite the ecological, economic, and cultural importance of macroalgae, we know very little about their mechanistic physiological responses to future CO_2_ enrichment, particularly compared to microalgae and terrestrial plants (Hibberd & Covshoff, 2010; Jungnick et al., 2014; Sandrini, Matthijs, Verspagen, Muyzer, & Huisman, 2014).

While field surveys have proved useful in identifying the general carbon uptake strategies of macroalgae, detailed knowledge of photosynthetic and growth responses to CO_2_ enrichment is unknown for most species and mainly focused on CCM species (Britton, Cornwall, Revill, Hurd, & Johnson, 2016; Fernández, Roleda, & Hurd, 2015; Israel & Hophy, 2002) and calcareous species (Hall‐Spencer et al., 2008; Jokiel et al., 2008). The effect of CO_2_ enrichment on non‐CCM species has been tested only twice, for *Lomentaria articulata* (Kübler, Johnston, & Raven, 1999) and *Amansia rhodantha* (Ho & Carpenter, 2017), with results being inconsistent between studies: *Lomentaria articulata* exhibited an increase in growth, while no response was detected for *Amansia rhodantha*. Likewise, for CCM species, both increased growth (Kim et al., 2016), no response (Fernández et al., 2015; Ho & Carpenter, 2017; Israel & Hophy, 2002; Rautenberger et al., 2015), and decreased growth have been reported (García‐Sánchez, Fernández, & Niell, 1994; Kim et al., 2016). Therefore, the question remains as to whether or not carbon uptake strategy can be used as a predictor of macroalgal responses to CO_2_ enrichment.

In this study, we compare the effects of CO_2_ enrichment on the growth, physiological, and biochemical responses of two temperate red macroalgae: a CCM species, *Lomentaria australis* (Kützing) Levring, and a non‐CCM species, *Craspedocarpus ramentaceus* (C. Agardh) Min‐Thein & Womersley. Both species are ecological dominants in the subtidal waters of eastern Tasmania, Australia. As this is only the third non‐CCM species to be included in a CO_2_ enrichment experiment, as well as the first study to compare biochemical responses of a non‐CCM species versus a CCM species, we aim to advance our understanding of the mechanisms that underlie macroalgal responses to CO_2_ enrichment. In addition, we conduct a comprehensive literature survey, including studies conducted prior to ocean acidification becoming a research field, to better understand the role that carbon uptake strategy plays in effecting the response of fleshy macroalgae to CO_2_ enrichment.

We hypothesized that: (a) CO_2_ enrichment will have no effect on the growth and photosynthetic rate of *L. australis* (CCM species), if the species used in this study has a high affinity for DIC. However, if the species has a low affinity for DIC, we predict a downregulation of energy‐costly uptake of HCO_3_^−^, with an increased passive CO_2_ uptake resulting in higher rates of growth and photosynthesis. (b) The net photosynthesis and growth of *C. ramentaceus* (non‐CCM species) will increase with CO_2_ enrichment, based on the likelihood that the species is currently carbon‐limited. (c) An elevated DIC availability will be used for the accumulation of storage compounds such as lipids in *L. australis* (CCM species), resulting in composition changes in lipid classes and fatty acids, whereas *C. ramentaceus* (non‐CCM species) will primarily invest in growth rather than storage of lipids.

## 2 MATERIALS AND METHODS

### 2.1 Macroalgal collection and culture maintenance

Twelve individual specimens of *Lomentaria australis* (a CCM species) and *Craspedocarpus ramentaceus* (a non‐CCM species) were collected from Tinderbox, Tasmania (S43°03'30.722 E147°19'52.583), on 21 October 2016 at 6 m depth using Scuba and were transported to the laboratory (30 min) in plastic bags containing seawater. Carbon uptake strategy of both species was confirmed by conducting pH‐drift experiments following Cornwall et al. (2015) (Supporting information Table S1). In the laboratory, the macroalgae were carefully rinsed with filtered seawater and visible epiphytes were gently removed. Prior to the experiment, individuals were acclimated to laboratory conditions in 20‐L aquaria for 48 hr, with constant aeration and a 12:12 light:dark photoperiod and a photon flux density (PFD) of 18–20 µmol photons m^−2^ s^−1^. During the pretreatment (24 hr) and experiment (7 days), the PFD was raised to 25–30 µmol photons m^−2^ s^−1^ which mimicked the PFD that was measured at the collection site at 6 m using integrating PAR sensors (Odyssey, Dataflow Systems Pty Ltd, Christchurch, New Zealand). In addition, light saturation curves were conducted to ensure that the light levels used in the experiment were sufficient for saturating photosynthesis (Supporting information Figure S1, Table S2). Seawater used in the experiment was collected south of Bruny Island, Tasmania, and was filtered to 1 μm and UV‐sterilized (Emperor Aquatics Smart HO UV Sterilizer, 025050‐2, 50 W lamp). The ammonium and nitrate concentrations were 0.46 and 0.20 µM, respectively, measured using a QuickChem 8500 Series 2 Automated Ion Analyzer (Lachat Instrument, Loveland, USA).

### 2.2 Experimental conditions

For each species, ~1 g of alga was randomly assigned to an individual container (650 ml) of an automated, pH‐controlled culture system. For both species, there were two experimental CO_2_ treatments: “current” (pH_T_ = 8.00, *p*CO_2_ = 365.17) and “future” (pH_T_ = 7.70, *p*CO_2_ = 1,015.24) at 12.5°C (detailed in the Supporting information Table S3). This corresponds to the local ambient pH/CO_2_ and the expected future seawater conditions, with *n* = 6 replicates for each species and CO_2_ treatment. The future pH was based on the RCP6.0, which is a “business as usual” modeling projection (IPCC, 2013). A magnetic stirrer in each container (set at 650 rpm) mixed the seawater to minimize the diffusion boundary layer around the blades of the macroalgae.

Target CO_2_ levels were achieved in each of the 24 culture containers using a system similar to that described in Bockmon, Frieder, Navarro, White‐Kershek, and Dickson (2013) with the modifications presented in Reidenbach et al. (2017). Briefly, every 4 hr, seawater in each 650‐ml culture container was replaced by flushing the container with ~1 L of seawater using Jebao DP‐4 Auto Dosing aquarium pumps. CO_2_ was added to this incoming seawater as it entered each culture container using a mixture of air and CO_2_, which was briefly in contact with seawater entering each culture container using a membrane contactor (Micromodule, model 0.5 × 1, Membrana, USA) placed near each culture container inlet. The contactors contain a microporous hollow fiber membrane, which allows efficient mixing of liquids and gases. Since the seawater and air/CO_2_ were in contact for only a few seconds, full equilibration of the air/CO_2_ mixture was not possible and air/CO_2_ ratios of approximately 400:1–1,000:1 were needed to achieved target CO_2_ levels in the seawater. These ratios were achieved using mass flow controllers (FMA5418A for air, FMA5402A for CO_2_; Omega Engineering, USA), whose flow rates were controlled by an analog output module housed in a USB chassis (NI9264 and cDAQ‐9174, National Instruments, USA). To maintain target CO_2_ levels, a feedback system monitored pH_T_ and adjusted the flow rates of the mass flow controllers automatically.

Seawater pH_T_ was measured using a modified version of the spectrophotometric pH_T_ system developed by McGraw et al. (2010). Briefly, a syringe pump and rotary valves were used to sample seawater (V6 pump, 24090 and 24493 valves, 23425 valve driver, Norgren, UK) while minimizing gas exchange. Spectra were acquired using an LED light source and a UV‐Vis spectrometer (BluLoop and USB2000+, Ocean Optics, USA) with a 1‐cm flow‐through quartz cuvette. Reference spectra were obtained from a 25 ml seawater sample; sample spectra were obtained by mixing 24.80 ml of seawater and 200 µl of 2 mM metacresol purple dye (857890, Sigma‐Aldrich, Australia) within the syringe pump. The temperature of each sample was recorded with a PT100 temperature sensor and a high‐precision data logger (PT‐104, PICO Technology, UK). All instrument control, spectra manipulations, and pH_T_ calculations were done using LabVIEW 2014 (National Instruments, USA).

pH_T_ was calculated from the temperature, salinity, and the absorbance spectra, which was calculated from the individual reference and sample spectra. To improve measurement precision, the absorbance at 434, 578, and 750 nm was determined using the quadratic fits of the absorbance spectra between 429–439 nm and 573–583 nm and a background signal averaged between 750 and 760 nm (McGraw et al., 2010). Each recorded measurement of pH_T_ was the average of four replicate measurements, which took ~2 min to obtain. The pH system was standardized using certified reference materials provided by Andrew Dickson, Scripps Institute for Oceanography, San Diego, USA (Dickson, Sabine, & Christian, 2007). A 0.03 pH unit offset was added to each measurement based on the difference between the measured pH and that calculated from the known DIC and A_T_ of the certified reference material. The appropriateness of this correction was verified through pH_T_ measurements of the Tris buffer.

### 2.3 Biotic responses

Growth rate was determined from changes in both thallus length and wet weight. Linear extension (distance from the apices to the main axis) was calculated from photographs of each individual placed on a grid on day 1 and day 7 using the software ImageJ (Schneider, Rasband, & Eliceiri, 2012). Relative growth rate (RGR) on a wet weight basis was calculated for each individual according to Yong, Yong, and Anton (2013): RGR = ln(*W_t_*/*W*_0_) × *t* ^−1^, where *W*_0_ is the wet weight at day 1 and *W_t_* is the final wet weight after 7 days. To remove surface water, blades were carefully blotted dry with tissue paper before weighing.

Net photosynthetic rates were measured at the end of the experiment (day 7) at the beginning of the light cycle in each culture tank under experimental conditions using an Orion RDO Probe 087010MD. Initial O_2_ measurements were conducted immediately after the culture tanks received a fresh seawater supply, and final O_2_ measurements were conducted two hours later. The probe was calibrated using a standard of 100% air saturation achieved by bubbling with air for 10 min. Oxygen production was calculated as final O_2_ measurements – initial O_2_ measurements and was standardized to tissue wet weight (g) per hour.

The maximum quantum yield (*F_v_/F_m_*) was used as an indicator of the performance of photosystem II (PSII) at the beginning and at the end of the experiment. *F_v_/F_m_* was measured using a pulse amplitude modulation (PAM) chlorophyll fluorescence meter (Diving PAM, Walz, Germany) using individuals that had been dark‐adapted for 10 min. The gain was set to 2, and *F*_0_ ranged between 200 and 1,000 for each measurement.

The content of photosynthetic pigments (chlorophyll *a* and phycobiliproteins) was analyzed using ~0.2 g (wet weight) of each individual on day 7. Chlorophyll *a* was extracted at room temperature according to Seely, Duncan, and Vidaver (1972) and Schmidt, Maraschin, and Bouzon (2010) using acetone and dimethylsulfoxide (DMSO). Pigment content was quantified spectrophotometrically with a S‐22 UV/Vis Spectrophotometer (Boeco, Germany), and concentrations (mg/g of wet weight) were calculated using the equations in Seely et al. (1972). To determine phycobiliprotein content (mg/g of wet weight), samples were flash frozen by immersion in liquid nitrogen and ground into a fine powder with a mortar and pestle. The extraction was carried out at 4°C using a 0.1 M phosphate buffer (pH = 7.2) according to the methods of Schmidt et al. (2010) and Sampath‐Wiley and Neefus (2007). All extractions were conducted in the dark.

Tissue samples from the apices were taken during the pretreatment and at the end of the experiment on day 7 from each individual sample to determine δ^13^C, C:N ratios, and lipid class and fatty acid content and composition. The samples were rinsed in Type I ultrapure water, frozen at −80°C, freeze‐dried (Labconco FreeZone 4.5), and ground with a mortar and pestle. δ^13^C, C and N content, and C:N were determined following the methods in Cornwall et al. (2015) using a NA1500 elemental analyzer coupled to a Thermo Scientific Delta V Plus via a ConFlo IV. Combustion and reduction were achieved at 1,020°C and 650°C, respectively. Values were normalized to the VPDB scale (Vienna Pee Dee Belemnite) via a 3‐point calibration using certified reference material. Both precision and accuracy were ±0.1 ‰ (1 *SD*). For the analyses, change in δ^13^C was calculated as the difference in δ^13^C between day 7 and day 1.

Total lipids were extracted following a modified version of Bligh and Dyer (1959) of the dried and weighed (ca. 40 mg) macroalgal tissue. Lipids were extracted overnight using a one‐phase methanol (MeOH): dichloromethane (DCM): Milli‐Q (2:1:08 v/v/v) solvent mixture. Phase separation was achieved by addition of 10 ml of DCM and 10 ml of Milli‐Q water the next day. The lower lipid‐containing layer was drained into a round bottom flask. After addition of few drops of MeOH, solvent was removed using a rotary evaporator (ca. 40°C). The lipid extracts were transferred to preweighed vials, and solvents were evaporated under a constant stream of nitrogen gas. The lipid‐containing vials were weighed for determination of total lipids. Samples were redissolved in DCM and stored at −20°C until further analysis.

To determine lipid class composition, an aliquot of the total lipid extract was spotted on SIII chromarods (5 µm particle size). Samples were co‐eluted with a standard mix to determine the lipid class composition including hydrocarbons (HC), steryl and wax esters (WE), triacylglycerols (TAG), sterols (ST), free fatty acids (FFA), and polar lipids which includes glycolipids and phospholipids (PL). The mobile phase consisted of hexane: diethyl ether (DEE): glacial acetic acid (GAA) (70:10:0.1 v/v/v). Chromarods were developed for 25 min and then dried at 100°C for 10 min. The dried rods were analyzed using an Iatroscan Mark V TH10 thin layer chromatography (TLC) with a flame ionization detector (FID). Peak identification was achieved by comparison with retention times of co‐eluted standards. For quantification, the SIC480II IatroscanTM integrating software (System Instruments, Mitsubishi Chemical Instruments) was used. The integrated areas were transformed to mass per µl spotted using pre‐determined linear regression calculations.

To analyze the fatty acids, an aliquot of the total lipid extract was saponified by addition of 2 ml of 5% KOH in 80% MeOH and placed on a heating block (80°C for 3 hr). After cooling, 1 ml of H_2_O was added and the mixture was extracted with hexane:DCM (4:1 v/v) to yield a nonsaponifiable neutral lipid fraction in the upper organic layer and free fatty acids in the lower aqueous layer. The aqueous layer was acidified by addition of 0.3 ml concentrated HCl and extracted three times with hexane:DCM (4:1, v/v). Solvents were removed under a stream of inert nitrogen gas, and methylation was performed by addition of MeOH:DCM:conc. HCl (10:1:1, v/v/v) and heating for 1 hr at 80°C. After cooling, 1 ml H_2_O was added and the resulting fatty acid methyl esters (FAME) were extracted three times into hexane:DCM (4:1, v/v). Solvents were removed under a stream of nitrogen gas. The samples were analyzed using gas chromatography (GC) using an Agilent Technologies 7890 (Palo Alto, California, USA) GC coupled with a flame ionization detector (FID) analyzer and equipped with a nonpolar EquityTM‐1 fused silica capillary column (15 m × 0.1 mm internal diameter, 0.1 µm film thickness). Agilent ChemStation software was used for quantification of FAME peaks. The individual fatty acids were identified using a GC–mass spectrometer (MS) using a Finnigan ThermoQuest GCQ GC‐MS System fitted with an on‐column injector and using ThermoQuest Xcalibur software.

To determine how macroalgae modify the carbonate chemistry of their local environment, seawater samples (13 ml) were collected from each replicate culture tank immediately after a change in seawater and again four hours later. These samples were collected both at day 1 and day 7. Simultaneously, pH_T_ was measured in the culture tanks with a Thermo Scientific Orion VERSA STAR 90 meter and pH electrode Orion 8107BNUMD Ross Ultra pH/ATC Triode, calibrated with a Tris buffer. Seawater samples were immediately poisoned with HgCl_2_ and stored under constant darkness. DIC concentrations of the water samples were measured using a DIC analyzer (Apollo SciTech DIC Analyzer Model AS‐C3) with an inbuilt CO_2_ analyzer (LI‐COR LI‐7000 CO_2_/H_2_O Analyzer). The CO_2_ analyzer was calibrated with a certified reference material provided by Andrew Dickson, Scripps Institute for Oceanography, San Diego, USA (Dickson et al., 2007). *A_T_* was calculated using the constants of Mehrbach, Culberson, Hawley, and Pytkowicz (1973), refitted by Dickson and Millero (1987).

### 2.4 Statistical analyses

All statistical analyses were performed using the software R 3.1.2 (R Core Team, 2016). A multivariate analysis of variance (MANOVA) was used to assess whether there were differences in biological responses (linear extension, wet weight RGR, Chl *a* and phycobiliprotein content, net photosynthesis, lipid content, fatty acid content, change in δ^13^C, change in C:N ratios, and change in carbon and nitrogen tissue content) between species and CO_2_ treatments. A second MANOVA was performed on the change in carbonate parameters ([H^+^], [HCO_3_^−^], [CO_3_^2−^], [CO_2_], and total DIC). The data passed the tests for MANOVA assumptions (normality and homogeneity of variance). When the interaction or main factor pH was significant (at α = 0.05), Tukey's honestly significant difference (THSD) post hoc tests were used to determine which treatments differed from each other (using the aov() and TukeyHSD() function in R). The Hedge's g is used as a measure of effect size (a g of 1 indicates that two groups differ by 1 *SD*). Hedge's g is calculated as follows: (M1‐M2)/SD_pooled_, where M1‐M2 is the difference in means of two groups and SD_pooled_ is the pooled standard deviation. Differences in *Fv/Fm* values were tested with a generalized linear model using the lme4 package, with treatment as fixed factor and start/end of the experiment as random factor (Bates, Mächler, Bolker, & Walker, 2015). A correspondence analysis (CA) was performed to detect differences in fatty acid composition between species and treatments using the Vegan package v.2.3‐5 (Oksanen et al., 2016). In the CA plot, objects (specimens) that are close to one another are likely to have a similar fatty acid composition. Specimens found near the point representing a fatty acid are likely to contain a higher relative level of that fatty acid. For this analysis, only fatty acids that were on average >0.5% of the TFA profile were used. Graphs were customized using the packages ggplot2 (Wickham, 2011), plyr (Wickham, 2011), and reshape2 (Wickham, 2007).

### 2.5 Literature synthesis

To further determine whether or not patterns in the responses of fleshy macroalgae to CO_2_ enrichment depend on their carbon uptake strategy, we conducted a comprehensive literature survey. We searched the databases Web of Science and Google Scholar using the keywords “CO_2_ enrichment”, “CO_2_ fertilization”, “ocean acidification”, “macroalgae”, and “seaweed” in various combinations. Fifty‐six studies published between 1989 and 2018 were analyzed, giving a total of 61 species. Studies were only examined if they met specific criteria. (a) The study had to report a growth response to CO_2_ enrichment (in addition, photosynthetic response and δ^13^C response were noted if those were reported); (b) the study needed to be a manipulative experiment comparing one or several macroalgal species in different *p*CO_2_ treatments (thus, we excluded field studies that were conducted in natural *p*CO_2_ gradients); (c) the study had to report whether the species used in the experiment were able to utilize HCO_3_^−^ or not (i.e., if the species possessed a CCM); (d) only studies on marine fleshy macroalgae were considered; (e) the macroalgae used in the experiment had to be identified to species level (i.e., no “turf”‐forming mats or community responses were included); (f) studies testing the effect of CO_2_ enrichment on microscopic early developmental stages and spore germination were excluded; and (g) the study had to be published in a peer‐reviewed journal. Many studies also tested additional experimental conditions (e.g., irradiance or nutrient concentrations), in combination with elevated *p*CO_2_. Those studies were included in our literature synthesis, but care was taken to only take into account the single‐term effects of elevated *p*CO_2_.

## 3 RESULTS

### 3.1 Growth

**Figure 1:**
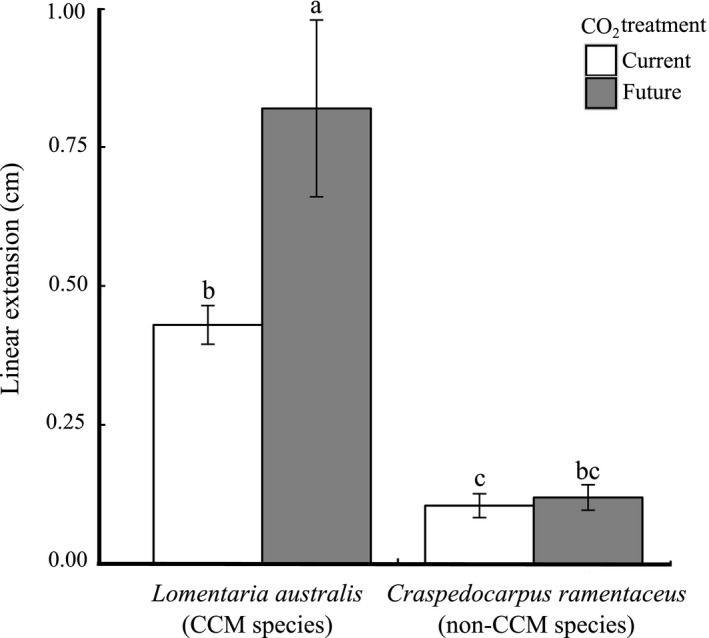
Linear extension (cm) of an algal species with carbon‐concentrating mechanism (*Lomentaria australis*; CCM species) and a species without (*Craspedocarpus ramentaceus*; non‐CCM species), with current (8.0) and future (7.7) CO_2_ treatment. Data are displayed as mean ± standard error, *n* = 6. Bars sharing a letter are not significantly different (Tukey's honestly significant difference tests, α = 0.05)

For *L. australis* (the CCM species), linear extension under the future CO_2_ treatment (pH = 7.70) was twofold greater (a Hedge's g effect size of 1.5) than the current conditions treatment (pH = 8.04) (Table 1, Figure 1), whereas for *C. ramentaceus* (the non‐CCM species), there was no significant effect of CO_2_ enrichment on growth (Table 1, Figure 1). Growth rates of *L. australis* under the future treatment were ca. eightfold and sevenfold greater (a Hedge's g effect size of 2.8) than *C. ramentaceus* under both current and future treatments, respectively (*p* < 0.0001 for both comparisons, Tukey's honestly significant difference tests). Growth rate on a wet weight basis showed a similar CO_2_ treatment effect (*p* = 0.095, Table 1; Supporting information Figure S2).

**Table 1:** Multivariate analysis of variance (MANOVA) table displaying *p*‐values and *F*‐values for all measured responses (linear extension, wet weight relative growth rate, net photosynthesis, chlorophyll a content, phycobiliprotein content, change in δ^13^C, change in C:N ratio, change in carbon tissue content, change in nitrogen tissue content, total lipid content, total fatty acid content, change in [H^+^], change in [HCO_3_^−^], change in [CO_2_], change in [CO_3_^2−^], and change in total DIC)

Response	Interaction		Factor			
			Species		CO_2_ treatment	
	*F‐*value	*p*‐value	*F*‐value	*p*‐value	*F*‐value	*p*‐value
Linear extension	**6.474**	**0.021**	–	–	–	–
Wet weight RGR	3.125	0.095	**15.743**	**0.001**	2.133	0.162
Chlorophyll a content (mg per g wet weight)	0.509	0.485	**20.563**	**<0.001**	0.169	0.686
Phycobiliprotein content (mg per g wet weight)	1.375	0.257	4.271	0.054	0.020	0.889
Net photosynthesis (µmol O_2_ hr^−^^1^ g^−^^1^)	1.813	0.196	1.385	0.256	0.075	0.788
Change in δ^13^C	1.229	0.283	**8.528**	**0.010**	**7.975**	**0.012**
Change in C:N ratio	0.274	0.608	1.350	0.261	0.083	0.777
Change in carbon tissue content	0.012	0.914	1.051	0.320	0.110	0.744
Change in nitrogen tissue content	0.031	0.861	**5.813**	**0.028**	0.140	0.713
Total lipid content	0.134	0.719	2.458	0.135	0.121	0.733
Total fatty acid content	0.220	0.645	**35.466**	**<0.001**	3.363	0.084
Change in [H^+^]	0.659	0.427	0.549	0.468	**133.993**	**<0.001**
Change in [HCO_3_^−^] (µmol/kg)	0.418	0.526	0.580	0.456	0.013	0.910
Change in [CO_2_] (µmol/kg)	0.501	0.488	0.395	0.537	**134.789**	**<0.001**
Change in [CO_3_] (µmol/kg)	0.025	0.877	1.671	0.212	0.171	0.684
Change in total DIC (µmol/kg)	0.754	0.396	0.119	0.734	2.572	0.125

Note

*p*‐values and *F*‐values of separate factors are not shown when the interaction between factors is significant (*α* = 0.05).

*p*‐values in bold have a significance of *p* < 0.05.

### 3.2 Net photosynthesis, pigments, and F_V_/F_M_

**Figure 2:**
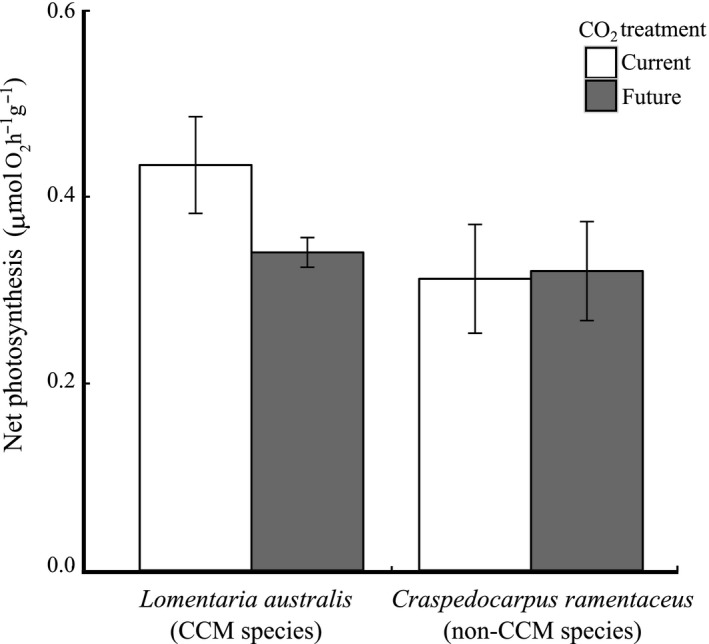
Net photosynthesis rates of a species with carbon‐concentrating mechanism (*Lomentaria australis*; CCM species) and a species without (*Craspedocarpus ramentaceus*; non‐CCM species), with current (8.0) and future (7.7) CO_2_ treatment. Data are displayed as mean ± standard error, *n* = 6. There were no significant differences between species and treatments

Net photosynthetic rates were similar for *L. australis* and *C. ramentaceus*, ranging from 0.3 to 0.4 µmol hr^−1^ g^−1^, and were unaffected by the CO_2_ treatment (Table 1, Figure 2). *C. ramentaceus* (the non‐CCM species) had a higher pigment content of both chlorophyll *a* and phycobiliproteins compared to *L. australis* (the CCM species), but pigment content was not affected by CO_2_ treatment (Supporting information Figure S3, Figure S4). The *F_v_*/*F_m_* value ranged between 0.51–0.54 and 0.49–0.58 for *L. australis* and *C. ramentaceus*, respectively, throughout the experiment and was not significantly different between treatments (*p* > 0.05 for all comparisons, linear mixed model Wald chi‐square test).

### 3.3 Stable isotopes and C:N ratios

**Figure 3:**
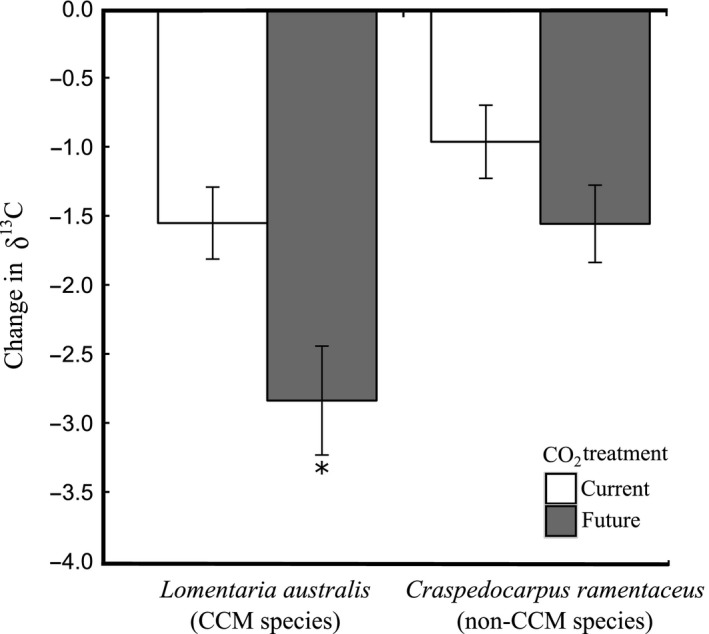
Change in δ^13^C ratios between day 1 (start of the experiment) and day 7 (end of the experiment) of a species with carbon‐concentrating mechanism (*Lomentaria australis*; CCM species) and a species without (*Craspedocarpus ramentaceus*; non‐CCM species), in current (8.0) and future (7.7) CO_2_ treatment. Data are displayed as mean ± standard error, *n* = 5–6 (with *n* = 5 for the ambient CCM treatment, and *n* = 6 for all other treatments). * denotes significantly different treatments (Tukey's honestly significant difference tests, α = 0.05)

*L. australis* (the CCM species) under the future treatment had a ca. twofold greater decrease (a Hedge's effect size of 1.2) in δ^13^C, compared to the individuals of the same species in current conditions (Figure 3, *p* < 0.05, Tukey's honestly significant difference test). CO_2_ treatment did not affect the degree of δ^13^C change of *C. ramentaceus* (the non‐CCM species) (*p* > 0.05, Tukey's honestly significant difference tests). C:N ratios increased from ~6.5 w/w at the start to ~8.5 w/w at the end of the experiment for each of the four treatment–species combinations, due to an increase in carbon content of the tissue (Supporting information Table S4). The magnitude of change in C:N ratio and C content did not differ between species and CO_2_ treatment (Table 1). The nitrogen content of the tissue remained relatively constant for both *L. australis* and *C. ramentaceus*, with less than 0.39% w/w difference from the start to end of the experiment (Supporting information Table S4).

### 3.4 Biochemical responses

**Figure 4:**
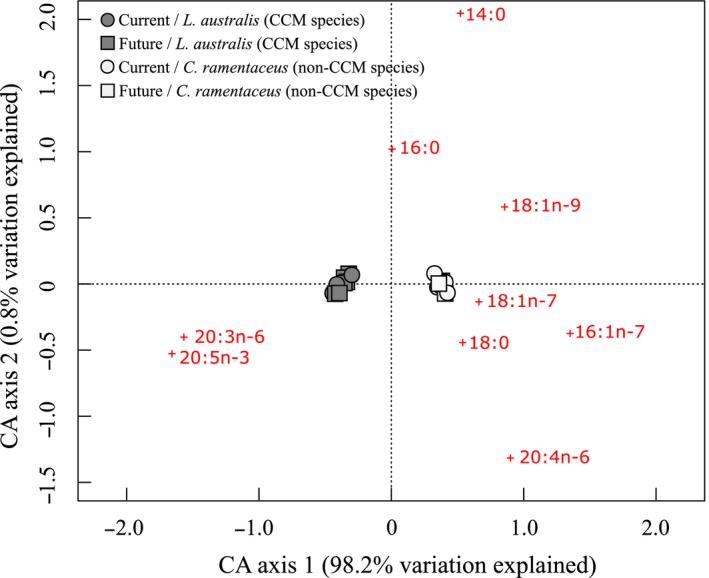
Correspondence analysis based on fatty acid composition of a species with carbon dioxide‐concentrating mechanism (*Lomentaria australis*; CCM species; gray) and a species without (*Craspedocarpus ramentaceus*; non‐CCM species; white), under current (circles) and future (squares) conditions. Crosses denote fatty acids. The nine most important fatty acids (the fatty acids that explain most of the variability) have been labeled

*L. australis* (the CCM species) had a higher total fatty acid content than *C. ramentaceus* (the non‐CCM species), but this was not affected by experimental treatment (Table 1; Supporting information Figure S5). Both species had similar total lipid content (Table 1; Supporting information Figure S6). Polar lipids dominated the lipid class composition of both *L. australis* and *C. ramentaceus* (Supporting information Figure S7). There were no differences in lipid class percentage between species and CO_2_ treatment (Supporting information Figure S7). A correspondence analysis (CA) showed that fatty acid composition differed significantly between species, but that the composition in each species did not change with CO_2_ treatment (Figure 4). The first CA axis explained 98.2% of the variation, and the second axis explained 0.8% of the variation. The difference in fatty acid composition was mainly driven by 16:1n‐7, 18:1n‐9, 18:0, 20:4n‐6, 20:3n‐6, and 20:5n‐3, where the former four fatty acids were more associated with *C. ramentaceus* and the latter two with *L. australis*.

### 3.5 Modification of seawater carbonate chemistry by algae

Over a period of four hours, the algae modified the pH, total DIC, CO_2_, HCO_3_^−^, and CO_3_^2−^ of the water in their culture tank, before they received a fresh supply of seawater (Supporting information Table S5). The [H^+^] decreased (i.e., pH increased), more so in the future treatments than in current treatments, but without differences between species (Table 1). The total DIC, HCO_3_^–^, and CO_2_ decreased, and the CO_3_^2−^ increased over the 4 hr. There were no significant differences between species and CO_2_ treatments for the increase in CO3^2−^ and the decrease in HCO_3_^–^ and total DIC. The decrease in pH and CO_2_ was greater in future conditions, but did not differ between species (Table 1).

### 3.6 Literature synthesis

Approximately 40% of the CCM species that were tested (21 out of 55 species) showed no growth response to CO_2_ enrichment, five species had decreased growth rates and 16 species increased growth with elevated CO_2_ (Table 2). For 13 CCM species, mixed responses were reported (i.e., several species were tested in multiple studies, and the responses reported were not concordant). Net photosynthetic rates either increased or did not respond to CO_2_ enrichment for the majority of the CCM species tested (11 and 10 species, respectively); in four species, photosynthesis decreased; nine species showed mixed responses; and for 21 species, photosynthetic responses were not reported (Table 2). Only three non‐CCM species (including our work) have been studied in relation to CO_2_ enrichment, with contrasting results in terms of both growth and net photosynthesis (Table 2). Overall, δ^13^C values were measured in only a minority of the studies published (10 out of 56 studies) (Table 2). None of the species increased δ^13^C values in response to elevated CO_2_, but no clear pattern in relation to growth and photosynthesis can be inferred.

**Table 2:** Response to CO_2_ enrichment reported in the literature for fleshy macroalgal species, regarding growth, photosynthesis, and δ^13^C values (“I” = increase, “D” = decrease, “NR” = no response, “–” = unreported)

Phylum and *species*	Enriched *p*CO_2_ level (μatm)	Response to CO_2_ enrichment			Putative carbon uptake strategy	Reference
		Growth	Photosynthesis	δ^13^C		
Rhodophyta (Red algae)						
*Amansia rhodantha*	~1,000	NR	–	–	Non‐CCM	[1]
*Chondrus crispus*	~800	NR & I	NR, D & I	–	CCM	[2]
*Craspedocarpus ramentaceus*	~1,000	NR	NR	NR	Non‐CCM	current study
*Gelidium crinale*	~750	NR	–	–	CCM	[3]
*Gracilaria chilensis*	650 & 1,250	I	I	–	CCM	[4]
*G. conferta*	~750	NR	–	–	CCM	[3]
*G. secundata*	–	I	–	–	CCM	[5]
*G. tenuistipitata*	–	D	D	–	CCM	[6]
*G. tikvahiae*	(pH = 6.0)	NR	I	–	CCM	[7]
Gracilariopsis lemaneiformis*	~700, ~1,000 & ~1,400	NR & I	NR & I	–	CCM	[8–12]
*Grateloupia cornea*	900 & 1,900	D	D	–	CCM	[13]
*Hypnea cornuta*	~750	NR	–	–	CCM	[3]
*H. musciformis*	~750	D	–	–	CCM	[3]
*H. spinella*	700 & 1,600	I	I	–	CCM	[14]
*Laurencia intricata*	~1,000	NR	I	D	CCM	[15]
*Lomentaria articulata*	700–1,800 (range)	I	–	D	Non‐CCM	[16]
*L. australis*	~1,000	I	NR	D	CCM	current study
*Melanothamnus harveyi*	~800 & ~1,500	NR & I	NR & I	–	CCM	[17]
*Palmaria palmata*	~1,000	NR & D	D	–	CCM	[18,19]
*Phycodrys rubens*	~1,000	NR	–	–	Possibly non‐CCM	[20]
*Plocamium cartilagineum*	900	D & I	–	–	CCM	[21]
*Porphyra linearis*	~750	D	NR	–	CCM	[22]
*Ptilota gunneri*	~1,000	NR	–	–	Possibly non‐CCM	[20]
*Pterocladiella capillacea*	~750	NR	–	–	CCM	[3]
*Pyropia haitanensis*	1,000	I	D & I	–	CCM	[23,24]
*P. leucosticta*	–	D	I	–	CCM	[25]
*P. yezoensis*	1,000 & 1600	I	I	–	CCM	[26]
Chlorophyta (Green algae)						
*Chaetomorpha linum*	(pH = 6.73)	I	–	–	CCM	[27]
*Cladophora coelothrix*	(pH = 6.73)	I	–	–	CCM	[27]
*C. patentiramea*	(pH = 6.73)	NR	–	–	CCM	[27]
*C. vagabunda*	(pH = 6.0)	I	I	–	Possibly non‐CCM	[7]
*Codium fragile*	900 & 1,900	NR	NR	–	CCM	[13]
*Monostroma grevillei var. arctica*	~1,000	NR	–	–	CCM	[20]
Ulva australis*	~900 & ~1,000 & ~1,900	NR & I	NR & I	–	CCM	[13,28–30]
*U. lactuca*	~700	NR	NR	–	CCM	[10,31]
*U. linza*	~750 & ~1,000	NR	D	–	CCM	[3,32]
*U. prolifera*	~1,000	I	NR	–	CCM	[33–35]
*U. pulchra*	–	I	–	–	CCM	[36]
*U. reticulata*	–	NR	–	–	CCM	[36]
U. rigida*	~1,200	NR & I	NR & D	NR	CCM	[36–39]
Ochrophyta (Brown algae)						
Alaria esculenta*	~1,000 & ~1,300	NR, D & I	NR	D	CCM	[20,40,41]
*Chnoospora implexa*	~1,000	NR	I	D	CCM	[15]
*Desmarestia aculeata*	~1,000 & ~1,300	D & I	NR & I	D & NR	CCM	[20,40,42]
*Dictyopteris undulata*	900	I	–	–	CCM	[21]
*Dictyota bartayresiana*	~1,000	NR	–	–	CCM	[1]
*Fucus vesiculosus*	~1,200	NR & D	–	–	CCM	[43–45]
*F. vesiculosus* f *mytili*	1,000	I	–	–	CCM	[46]
*Laminaria solidungula*	~1,200	NR	NR	NR	CCM	[47]
*Lobophora variegata*	~1,000	NR	–	–	CCM	[1]
*Macrocystis pyrifera*	~1,200	NR	NR	NR	CCM	[48]
*Nereocystis luetkeana*	~3,000	I	I	–	CCM	[49,50]
*Padina pavonica*	~750	NR	–	–	CCM	[3]
*Saccharina japonica*	~1800	NR	I	–	CCM	[51]
S. latissima*	~1,000 & ~1,200 & ~3,000	NR, D & I	NR & I	NR & D	CCM	[18,41,47,49,52]
*Saccorhiza dermatodea*	~1,000	I	–	–	CCM	[20]
Sargassum fusiforme*	~700 & ~1,000	D & I	NR & I	–	CCM	[53–55]
*S. horneri*	900 & 1,900	NR & I	NR	–	CCM	[13,21]
*S. muticum*	1,000	I	I	–	CCM	[56]
*S. thunbergii*	900 & 1,900	I	I	–	CCM	[13]
*S. vulgare*	~750	NR	–	–	CCM	[3]
*Turbinaria ornata*	~1,000	NR	NR	NR	CCM	[15]

Notes

The elevated *p*CO_2_ level (which was compared to ambient control levels in each study), the carbon uptake strategy (non‐CCM or CCM), and references are also noted. * indicates species of which detailed physiological and biochemical regulatory mechanisms are known.

References: [1] Ho and Carpenter (2017); [2] Sarker, Bartsch, Olischläger, Gutow, and Wiencke (2013); [3] Israel and Hophy (2002); [4] Gao et al. (1993); [5] Lignell and Pedersén (1989); [6] García‐Sánchez et al. (1994); [7] Rivers and Peckol (1995); [8] Chen, Zou, Zhu, and Yang (2017); [9] Xu, Zou, and Gao (2010); [10] Liu, Zou, and Yang (2018); [11] Zou and Gao (2009); [12] Kang, Kambey, Shen, Yang, and Chung (2017); [13] Kim et al. (2016); [14] Suárez‐Álvarez, Gómez‐Pinchetti, and García‐Reina (2012); [15] Bender‐Champ, Diaz‐Pulido, and Dove (2017); [16] Kübler et al. (1999); [17] Olischläger and Wiencke (2013); [18] Nunes et al. (2016); [19] Sebök, Herppich, and Hanelt (2017); [20] Gordillo, Carmona, Viñegla, Wiencke, and Jiménez (2016); [21] Kram et al. (2016); [22] Israel, Katz, Dubinsky, Merrill, and Friedlander (1999); [23] Liu and Zou (2015a); [24] Xu, Chen, et al. (2017); [25] Mercado, Javier, Gordillo, Xavier Niell, and Figueroa (1999); [26] Gao et al. (1991); [27] de Paula Silva, Paul, Nys, and Mata (2013); [28] Reidenbach et al. (2017); [29] Kang and Chung (2017); [30] Kang and Kim (2016); [31] Liu and Zou (2015b); [32] Gao et al. (2018); [33] Xu and Gao (2012); [34] Li, Xu, and He (2016); [35] Li, Zhong, Zheng, Zhuo, and Xu (2018); [36] Björk, Haglund, Ramazanov, and Pedersén (1993); [37] Gordillo, Niell, and Figueroa (2001); [38] Rautenberger et al. (2015); [39] Gordillo, Figueroa, and Niell (2003); [40] Iñiguez et al. (2016a); [41] Gordillo et al. (2015); [42] Iñiguez, Heinrich, Harms, and Gordillo (2017); [43] Gutow et al. (2014); [44] Kawamitsu and Boyer (1999); [45] Ober and Thornber (2017); [46] Mensch et al. (2016); [47] Iñiguez et al, (2016b); [48] Fernández et al. (2015); [49] Swanson and Fox (2007); [50] Thom (1996); [51] Kang and Chung (2018); [52] Olischläger, Iñiguez, Koch, Wiencke, and Gordillo (2017); [53] Zou (2005); [54] Zou, Gao, and Luo (2011); [55] Jiang, Zou, Lou, and Gong (2018); [56] Xu, Gao, Gao, Xu, and Wu (2017).

## 4 DISCUSSION

Carbon uptake strategy has been a key concept in developing hypotheses related to how fleshy macroalgae will respond to CO_2_ enrichment (Gutow et al., 2014; Hepburn et al., 2011; Ho & Carpenter, 2017; Hurd, Lenton, Tilbrook, & Boyd, 2018; Israel & Hophy, 2002; Johnson, Comeau, Lantz, & Smith, 2017; Koch et al., 2013). The aim of this study was to compare the physiological and biochemical responses of *L. australis* (a CCM species) with *C. ramentaceus* (a non‐CCM species) to CO_2_ enrichment in order to broaden our knowledge on whether or not carbon uptake strategy is a useful predictor of the responses of fleshy macroalgae to CO_2_ enrichment. Our results, along with a careful scrutiny of the literature, reveal that fleshy macroalgal species exhibit a wide array of responses to CO_2_ enrichment in terms of both growth and photosynthesis (Table 2). As these responses also vary between species with the same carbon uptake strategy, we suggest that it is not currently possible to predict the responses of macroalgae to a higher CO_2_ ocean based solely on the presence/absence or type of CCM: A greater mechanistic understanding is needed before such predictions can be made.

### 4.1 The response of CCM species

For CCM species that responded positively to CO_2_ enrichment, the range of growth responses varied from a ~ 1.2‐fold increase in *Ulva australis* (Kim et al., 2016) to a ~ 4‐fold increase in *Gracilaria chilensis* (Gao, Aruga, Asada, & Kiyohara, 1993)**,** fitting well with the twofold response of *Lomentaria australis* (the CCM species in our study). However, it is noteworthy that mixed growth responses to CO_2_ enrichment were reported for 13 of the CCM species and mixed photosynthetic responses for nine of the CCM species included in our literature survey (Table 2), as several species have been tested in multiple studies and the results did not always match. This inconsistency is most likely because other environmental factors also affect growth and physiological responses. For example, the kelp *Saccharina latissima* showed either no response or increased growth rates to elevated *p*CO_2_, depending on levels of UV radiation (Gordillo, Aguilera, Wiencke, & Jiménez, 2015). In another study, *S. latissima* had decreased growth rates in response to CO_2_ enrichment (Swanson & Fox, 2007), but the *p*CO_2_ used (~3,000 µatm) was much higher (3×) than that typically used in OA experiments and may be unrealistic, as the corresponding decrease in pH is larger than the worst‐case IPCC scenario (RCP8.5; IPCC, 2013). Overall, our synthesis indicates that many (~40%) CCM species are likely to be saturated for inorganic carbon, but that the benefits of CO_2_ enrichment for those that are not saturated for inorganic carbon could be substantial in terms of increased growth and productivity.

The reason for the decreased growth rate recorded for five CCM species with CO_2_ enrichment is unknown, but may be due to a sensitivity to increasing H^+^ concentrations. This sensitivity is known to exist in calcifying organisms (e.g., coccolithophores and zooplankton), as elevated H^+^ directly decreases calcification, and in fish, where increasing H^+^ causes acidosis and behavioral changes (Hurd et al., 2018). As H^+^ is key in regulating cellular homeostasis, changing H^+^ concentrations could affect macroalgal metabolic processes and CCM activity. The effect of increasing H^+^ concentrations has not been addressed for fleshy macroalgae, with the exception of Roleda, Morris, McGraw, and Hurd (2012), who found an interactive effect of pH (i.e., H^+^) and DIC on early microscopic life‐history stages of *Macrocystis pyrifera*. As H^+^ concentrations will increase by 200% by 2100 (RCP6.0 scenario; IPCC, 2013), this calls for future research on how increasing H^+^ concentrations, and the interaction with DIC enrichment, affect macroalgal physiology.

The increase in growth rate of *Lomentaria australis* with elevated CO_2_ most likely occurred as a result of the downregulation of an energy‐costly CCM. This idea is supported by the greater shift to more negative δ^13^C values under future CO_2_ conditions. The increase in tissue carbon content for all treatments and lack of change in lipid accumulation both indicate that the decrease in δ^13^C values is not related to sugar use or lipid accumulation, but was caused by a change in carbon uptake strategy and the relative use of HCO_3_^−^ and CO_2_. As with growth rates and net photosynthesis, mixed results were reported for δ^13^C in the current literature (Table 2). For example, an increased growth rate was combined with decreased δ^13^C in *Lomentaria australis* (current study), whereas growth rates and δ^13^C simultaneously decreased in *Desmarestia aculeata* (Iñiguez et al., 2016a). This shows that it is important to measure physiological processes and parameters (e.g., net photosynthetic rates, C:N ratios, and δ^13^C) in addition to measuring the response (i.e., growth), if we are to understand how the response to CO_2_ enrichment is regulated.

### 4.2 The response of non‐CCM species

The lack of response of *Craspedocarpus ramentaceus* to CO_2_ enrichment suggests that growth and photosynthesis were saturated at current CO_2_ levels, as there was no evidence of nitrogen or light limitation in this experiment. C:N ratios were typical of N‐sufficient Rhodophyta (Atkinson & Smith, 1983; Supporting information Table S4), the growth rates of the specimens in our experiment are within the healthy range of fleshy Rhodophyta (Kübler et al., 1999; Nishihara, Terada, & Noro, 2004), and the light levels were sufficient for saturating photosynthesis (Supporting information Figure S1, Table S2). Only three non‐CCM species (including our work) have been studied in relation to CO_2_ enrichment, with contrasting results (Table 2). This indicates that, as with CCM species, non‐CCM species could have different affinities for CO_2_. For non‐CCM species, the affinity for DIC will mainly depend on Rubisco activity and kinetics (as this is the enzyme involved in carbon fixation). The amount of Rubisco, the *V*_max_ (maximal Rubisco activity) and *K_m_* (affinity of Rubisco for CO_2_) vary greatly between species (Israel & Hophy, 2002). Red algae, for example, have a higher affinity for CO_2_ relative to O_2_ compared to green and brown algae, and, in general, studies show that Rubisco enzymes in CO_2_‐only users have evolved a higher affinity for CO_2_ than species with a CCM (Badger et al., 1998). In addition, environmental conditions play an important role in Rubisco kinetics. High temperatures, for example, decrease the affinity for CO_2_ in algae (Beardall, Stojkovic, & Larsen, 2009) and the activation state of Rubisco changes with light and CO_2_ concentrations, as is also known from terrestrial plants (Salvucci & Crafts‐Brandner, 2004). The capacity of non‐CCM species to fix carbon is often thought to be adapted to low rates of light absorption, in order to avoid photodamage (Maberly et al., 1992), and it is possible that non‐CCM species are therefore unable to take advantage of elevated CO_2_, rather than being limited in carbon. Studying how Rubisco kinetics change with CO_2_ enrichment could give more insight into the response of non‐CCM species. Given that 35% of red macroalgae are non‐CCM (Kübler & Dudgeon, 2015), we emphasize the need to incorporate more non‐CCM species in future laboratory studies if we are to determine how macroalgae will respond to future global change.

### 4.3 Biochemical responses

Our study provides the first findings on the impacts of CO_2_ enrichment on the lipid content, lipid class, and fatty acid composition in a CCM species compared to a non‐CCM species. Both species invested in growth rather than storing lipids, which supported our hypothesis for *C. ramentaceus* (the non‐CCM species), but did not support the hypothesis that lipid content and fatty acid composition would change in *L. australis* (the CCM species). There are several possible explanations for the lack of biochemical response of *L. australis*: (a) Previous work, mainly on microalgae, has shown that it is the combination of excess carbon and limiting nitrogen (i.e., insufficient nitrogen for protein synthesis) that provides optimal conditions for an increase in carbon flow toward acetyl coenzyme A (acetyl Co‐A) and NADPH for FA synthesis (Sun et al., 2016). However, the nitrogen concentration used in this experiment was not likely to be limited, as the concentrations were that of ambient seawater and C:N ratios in the algal tissue were in the range consistent with nitrogen sufficiency (Atkinson & Smith, 1983). (b) Results from the lipid class composition showed that most of the lipid occurred as structural polar lipids. This indicates that both species accumulate storage lipids only in very low amounts. The ability to accumulate storage lipids might be exhibited only by certain macroalgal taxa, while others, including some red algae, generally exhibit low storage lipid concentrations and predominantly change the composition and concentration of structural polar lipids and therefore no changes were observed (Schmid, Guihéneuf, & Stengel, 2017).

### 4.4 DIC limitation as a predictor of macroalgal responses to future high CO_2_ oceans

**Figure 5:**
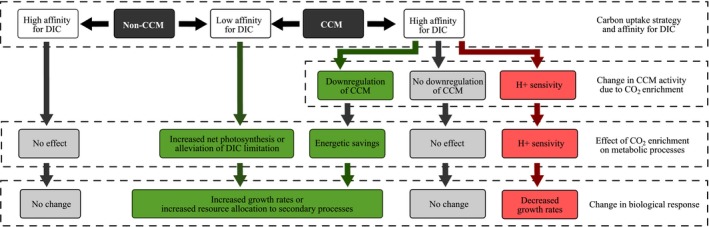
Predicted physiological and growth response of fleshy macroalgae to CO_2_ enrichment based on their carbon uptake strategy and affinity for DIC. Species with a low affinity for DIC are likely to be limited in DIC under current conditions, and species with a high affinity are likely to be saturated for DIC. There is literature evidence that some CCM species are sensitive to increased H^+^ concentrations, illustrated on the right hand side of the figure: Although not illustrated, H^+^ sensitivity for non‐CCM species may also be possible. This figure builds on that of Cornwall et al. (2017)

The results from this study and prior studies (Table 2) highlight that—even though carbon use strategy will play a role—the responses of macroalgae to CO_2_ enrichment cannot be inferred solely from the carbon uptake strategy as has been implicated in field studies (Cornwall et al., 2017, 2015 ; Diaz‐Pulido et al., 2016; Hepburn et al., 2011). We suggest that the key to predicting the response(s) of macroalgae to a future high CO_2_ ocean is to understand which species have growth rates that are limited by DIC availability under current CO_2_ levels, and which species are saturated. In addition, the ability of CCM species to downregulate their CCM will be a decisive factor in their response to future CO_2_ enrichment conditions. This view is summarized in Figure 5, which synthesizes the predicted responses of fleshy macroalgae based on their putative limitation by DIC (with high‐affinity species being saturated and low‐affinity species being limited) and carbon uptake strategy, expanding on the figure of Cornwall et al. (2017). We additionally suggest that some species may be sensitive to higher [H^+^] also predicted for the future, and a mechanistic understanding of the interactions between DIC and [H^+^] is needed (Bach et al., 2013; Roleda et al., 2012). To be able to predict the responses of macroalgae to CO_2_ enrichment, future studies should focus on providing a better understanding of the physiological mechanisms that underlie DIC acquisition: Details of physiological and biochemical regulatory mechanisms are known for only a few of the studied species listed in Table 2 (indicated with *), and these are mostly species with high commercial value or fouling species that inhibit growth of species with commercial value. This emphasizes an urgent need for targeted physiological experiments, as well as molecular studies, on a wide range of macroalgal species from the tropics to the poles, if we are to gain a mechanistic understanding of macroalgal responses to a future higher CO_2_ world, and move beyond predictions based on field observational studies.

## DATA ACCESSIBILITY STATEMENT

All data created during this research are available at the IMAS data management system and at FigShare (https://doi.org/10.6084/m9.figshare.7189382 and https://doi.org/10.6084/m9.figshare.7189292).

## CONFLICT OF INTEREST

None declared.

## AUTHOR CONTRIBUTION

L.M.L. carried out laboratory experiment and analyzed the data with assistance by M.S, P.P.L., and D.B. L.M.L., M.S, P.P.L., and C.L.H. designed the study. L.M.L., M.S., and C.L.H. wrote the original draft, with subsequent contributions by all other authors. A.T.R., P.V., P.D.N., and C.M.M. collected laboratory data, contributed materials, and assisted with carbonate chemistry calculations.
