# Supplementary material for: Responses of macroalgae to CO2 enrichment cannot be inferred solely from their inorganic carbon uptake strategy
Source: Ecol Evol. 2018 Dec 14;9(1):125–40. doi: 10.1002/ece3.4679 (PMC6342131; doi:10.1002/ece3.4679)
Supplement: Supplementary file 1 [file ECE3-9-125-s001.docx]

**Supplementary information for:**

Responses of macroalgae to CO_2_ enrichment cannot be inferred solely from their inorganic carbon uptake strategy

Luna M. van der Loos^1,2*^, Matthias Schmid^1^, Pablo P. Leal^1,3^, Christina M. McGraw^4^, Damon Britton^1^, Andrew T. Revill^5^, Patti Virtue^1,5,6^, Peter D. Nichols^1,5^, Catriona L. Hurd^1^

^1^Institute for Marine and Antarctic Studies, University of Tasmania, Hobart, Tasmania 7001, Australia

^2^University of Groningen, Marine Ecology, Nijenborgh 7, 9747, AG Groningen, The Netherlands

^3^Instituto de Fomento Pesquero (IFOP), Balmaceda 252, Puerto Montt, Casilla 665, Chile

^4^Department of Chemistry, NIWA/University of Otago Research Centre for Oceanography, University of Otago, Dunedin, 9016, New Zealand

^5^CSIRO Oceans and Atmosphere, Hobart, Tasmania 7000, Australia

^6^Antarctic Climate and Ecosystems, Cooperative Research Centre, Hobart, Tasmania 7000, Australia

*Corresponding author, email: lunavdloos@gmail.com; phone: +31 6 34 29 00 36

**Supplementary information: pH drift experiments**

pH drift experiments are used to determine which species possess a carbon concentrating mechanism (CCM) and which species do not, and were conducted in order to select species to use in the experiment. Macroalgae increase the pH of their own environment through photosynthesis. The final pH value to which an alga can increase the seawater is called the “pH compensation point”. At pH_NBS_ 9.0, CO_2_ is functionally absent. Macroalgae with a compensation point higher than 9 are considered to possess a CCM.

Two red macroalgae species, *Lomentaria australis* (Kützing) Levring and *Craspedocarpus ramentaceus* (C.Agardh) Min-Thein & Womersley, that were considered by Cornwall et al. (2015) to be CCM and non-CCM, respectively, were sampled at Tinderbox, Tasmania (S43°03'30.722 E147°19'52.583), at 6 m. The specimens were transported to the laboratory (30 min) in plastic bags containing seawater. In the laboratory, the macroalgae were carefully rinsed with seawater and visible epiphytes were removed. Prior to the pH drift experiment, individuals were acclimated to lab conditions in aquaria for 24 hours, with constant aeration and a 12:12 light:dark photoperiod. Day light intensity was 18-20 µmol photons m^-2^ s^-1^. Approximately 1 g of algae was placed in 50-ml transparent plastic containers with seawater. Seawater had a mean pH_NBS_ of 8.17 and a salinity of 37‰. Where possible, three replicates of each species were used. Seawater pH was measured with a Thermo Scientific Orion VERSA STAR 90 metre and pH electrode Orion 8107BNUMD Ross Ultra pH/ATC Triode, calibrated with pH 7.0 and 10.0 NBS buffers. The containers were randomly ordered on a benchtop orbital shaker under 250 mol photons m^-2^ s^-1^ at 12 °C. After 24 hours, the final pH was measured. This is the optimal time according to Hepburn et al. (2011).

The results of the pH drift experiment can be found in Table S1.

**Table S1**. Results of pH drift experiment.

| **Species** | **Sample location** | **Sample depth** | **pH after 24h** | **Carbon uptake mechanism** |
| --- | --- | --- | --- | --- |
| *Lomentaria australis* (Kützing) Levring | Tinderbox | 6m | 9.376 | CCM |
| *Craspedocarpus ramentaceus* (C.Agardh) Min-Thein & Womersley | Tinderbox | 6m | 8.866 | non-CCM |

**Supplementary information: light curves**

A PE curve was made for both species used in this study (*Lomentaria australis* and *Craspedocarpus ramentaceus*) prior to the start of the experiment using a Pulse Amplitude Modulation (PAM) chlorophyll fluorescence meter (Diving PAM, Walz, Germany). All individuals had been dark-adapted for 10 minutes. The gain was set to 2 and F0 ranged between 200-1000 for each measurement. The relative electron transport rate was calculated by multiplying the yield (F_v_/F_m_) with the PAR (photosynthetically active radiation). Subsequently, the data was fitted using the package phytotools() in R and the curve by Webb et al. (1974). From the fitted curves, the α value (slope of the curve) and E_k_ value (light saturation parameter) were estimated. The E_k_ is the transition zone between unsaturated and saturated photosynthesis and therefore gives an indication of the light acclimation of the specimen; a low E_k_ means the specimen is low-light acclimated and a high E_k_ means the specimen is high-light acclimated.

As evident from Figure S1 and Table S2, the curves are relatively steep (high α) and are quickly saturated (reaching P_max_), which results in a low E_k_ value for both species. This indicates that both species were low-light acclimated.

**Table S2.** The α value and E_k_ value (light saturation parameter) of a species with carbon concentrating mechanism (*Lomentaria australis*; CCM species) and a species without (*Craspedocarpus ramentaceus*; non-CCM species). Data are displayed as mean ± standard error.

| species | α ± SE | E_k_ ± SE |
| --- | --- | --- |
| *L. australis* (CCM) | 0.10 ± 0.02 | 23.70 ± 4.24 |
| *C. ramentaceus* (non-CCM) | 0.08 ± 0.01 | 31.55 ± 5.06 |

**
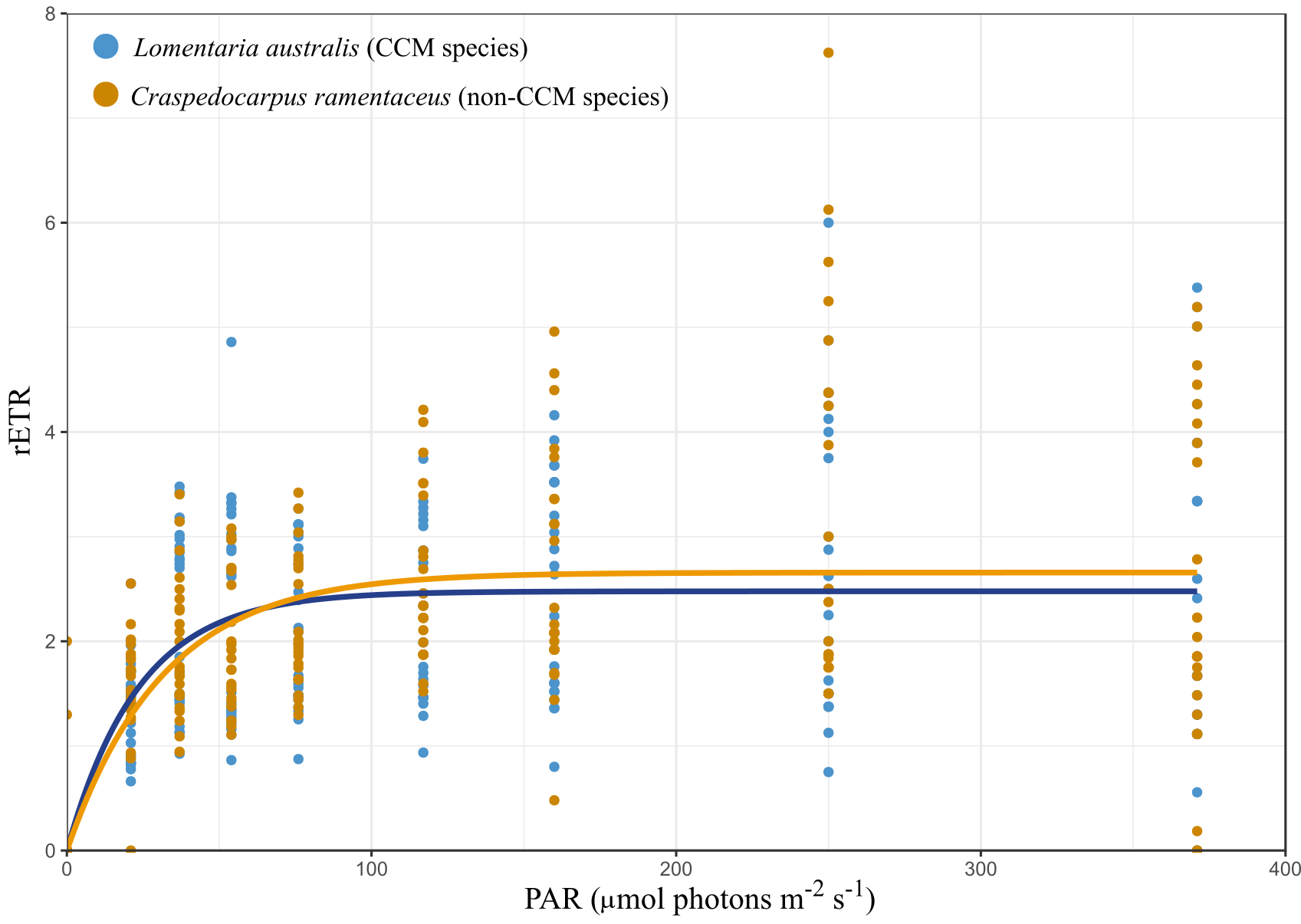
**

**Figure S1.** PE curves from Diving PAM, with rETR on the y-axis (defined as yield * PAR) and on the x-axis the photosynthetically active radiation (in µmol photons m^-2^ s^-1^), of a species with carbon concentrating mechanism (*Lomentaria australis*; CCM species; blue) and a species without (*Craspedocarpus ramentaceus*; non-CCM species; yellow). The points depict individual measurements and the solid lines are fitted curves (using the formula supplied by Webb et al. 1974)

**Supplementary information: experimental conditions**

The pH_T,12.5C_ of the current treatment was 8.04 (8.03, 8.06; ± standard deviation, N = 379); the pH_T,12.5C_ of the future treatment was 7.70 (7.68, 7.73; N = 356) (Table S3). This variability was larger than the precision of the measurement (0.005 pH units), which was determined from eight consecutive measurements made at the same temperature. The temperature was maintained at 12.49 °C ± 0.04 °C (± standard error, N = 192); the salinity was 37‰.

**Table S3.** Seawater carbonate chemistry within culture tanks. Carbonate parameters were calculated from pH (n = 735) and DIC (dissolved inorganic carbon) measurements (n = 96) of seawater corresponding to each CO_2_ treatment. Measurements were standardised to a temperature of 12.5 °C. Values in parentheses are standard deviations for pH and standard error for other carbonate parameters.

| **Carbonate chemistry** | **CO_2_ treatment** | |
| --- | --- | --- |
|  | **Current** | **Future** |
| pH_T,12.5°C_ | 8.04 (8.03, 8.06) | 7.70 (7.68, 7.73) |
| DIC (µmol kg^-1^) | 2106.24 (1.36) | 2246.33 (4.44) |
| A_T_ (µmol kg^-1^) | 2341.24 (2.39) | 2320.71 (2.05) |
| HCO_3_^-^ (µmol kg^-1^) | 1927.30 (1.51) | 2130.43 (4.30) |
| CO_3_^2-^ (µmol kg^-1^) | 164.39 (1.13) | 75.43 (1.60) |
| pCO_2_ (µatm) | 365.17 (2.81) | 1015.24 (47.62) |

**Supplementary Figures:**

**
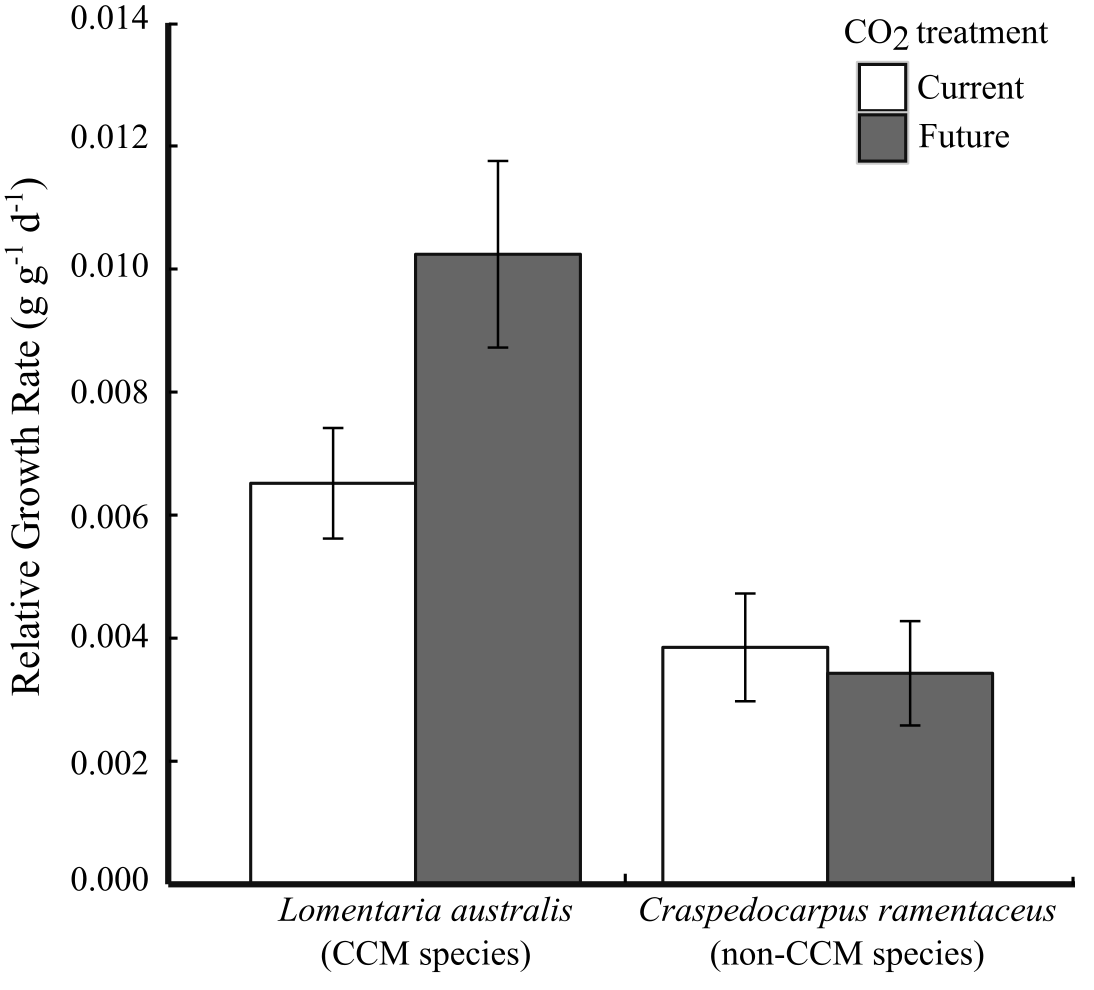
**

**Figure S2.** Relative growth rate (g g^-1^ d^-1^) based on wet weight, of a species with carbon concentrating mechanism (*Lomentaria australis*; CCM species) and a species without (*Craspedocarpus ramentaceus*; non-CCM species), with current (8.0) and future (7.7) CO_2_ treatment. Data are displayed as mean ± standard error, n = 5-6.


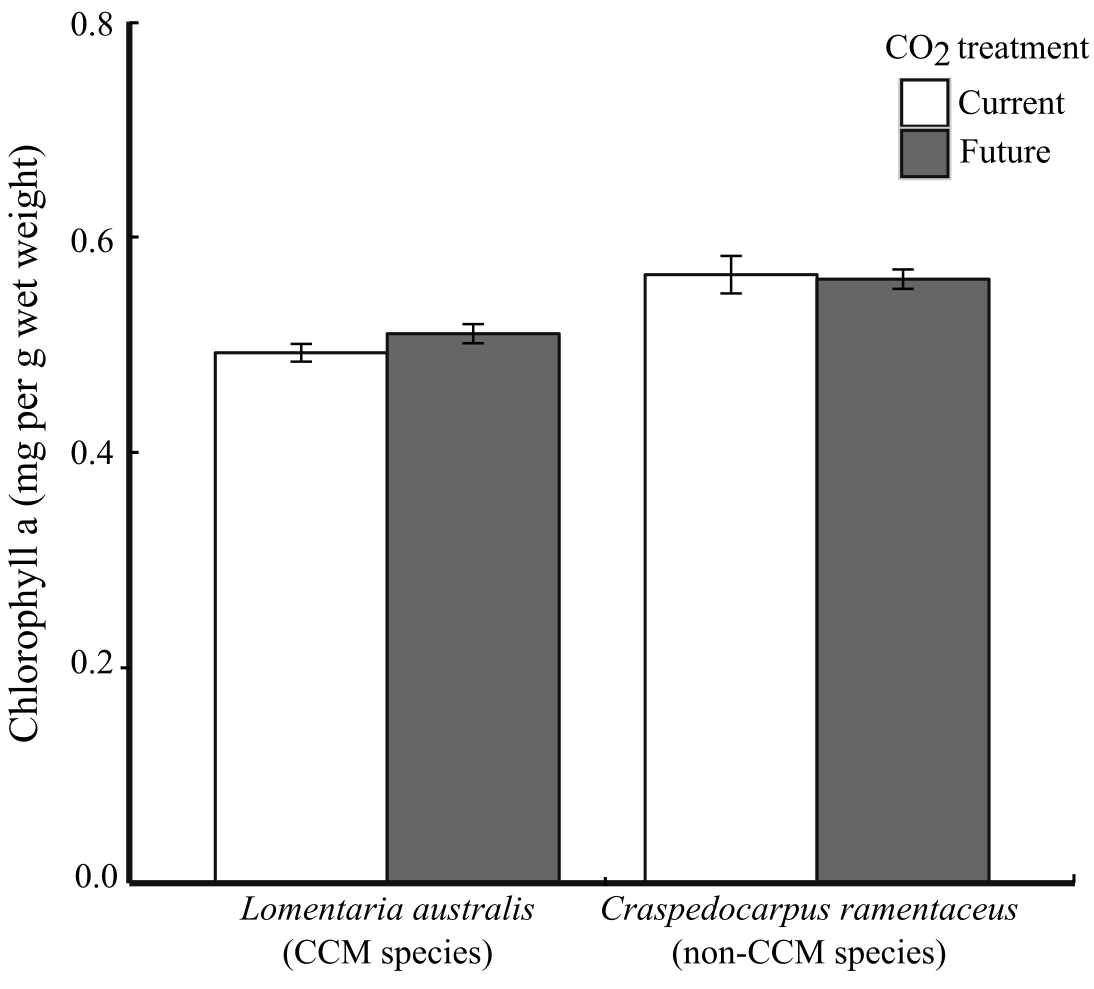


**Figure S3.** Chlorophyll a content of a species with carbon concentrating mechanism (*Lomentaria australis*; CCM species) and a species without (*Craspedocarpus ramentaceus*; non-CCM species), with current (8.0) and future (7.7) CO_2_ treatment. Data are displayed as mean ± standard error, n = 6.


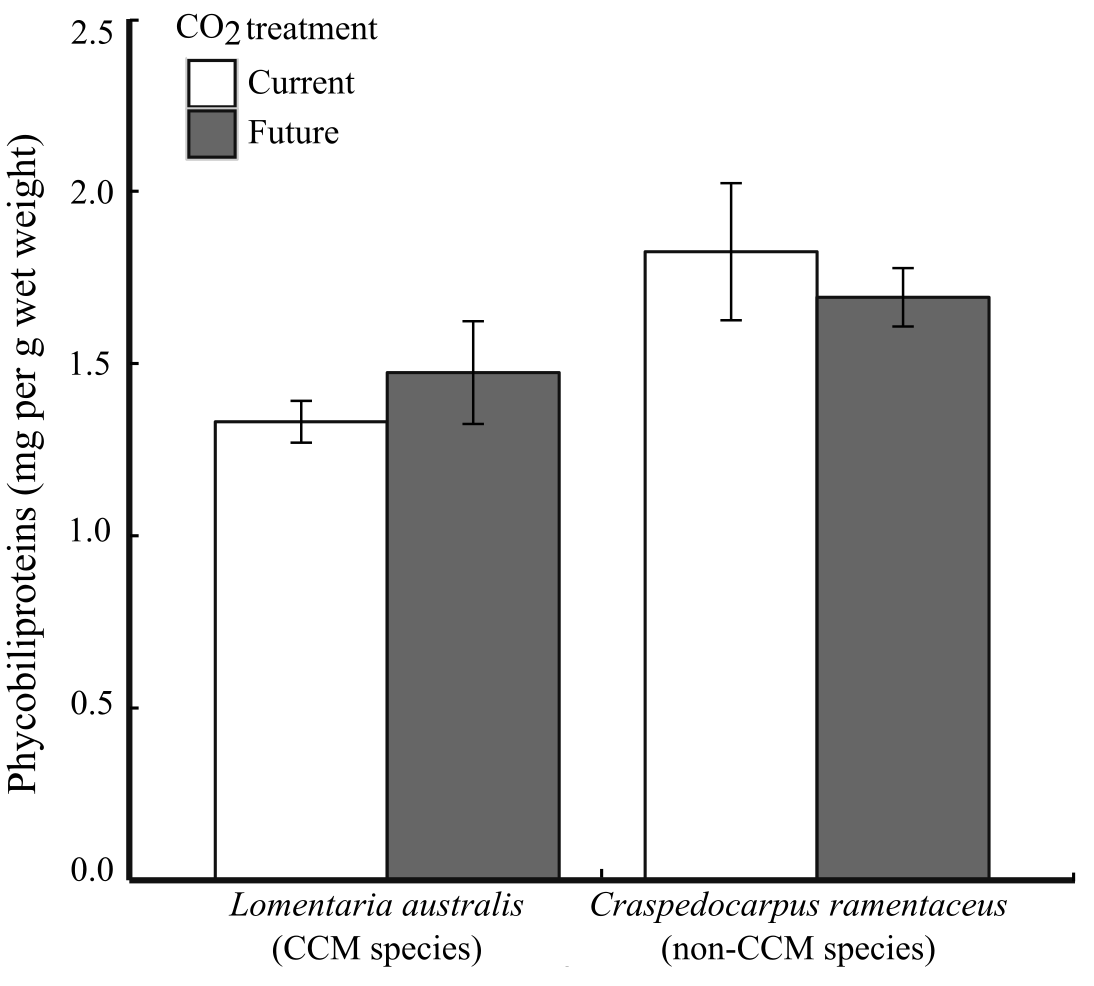


**Figure S4.** Phycobiliprotein content of a species with carbon concentrating mechanism (*Lomentaria australis*; CCM species) and a species without (*Craspedocarpus ramentaceus*; non-CCM species), with current (8.0) and future (7.7) CO_2_ treatment. Data are displayed as mean ± standard error, n = 6.


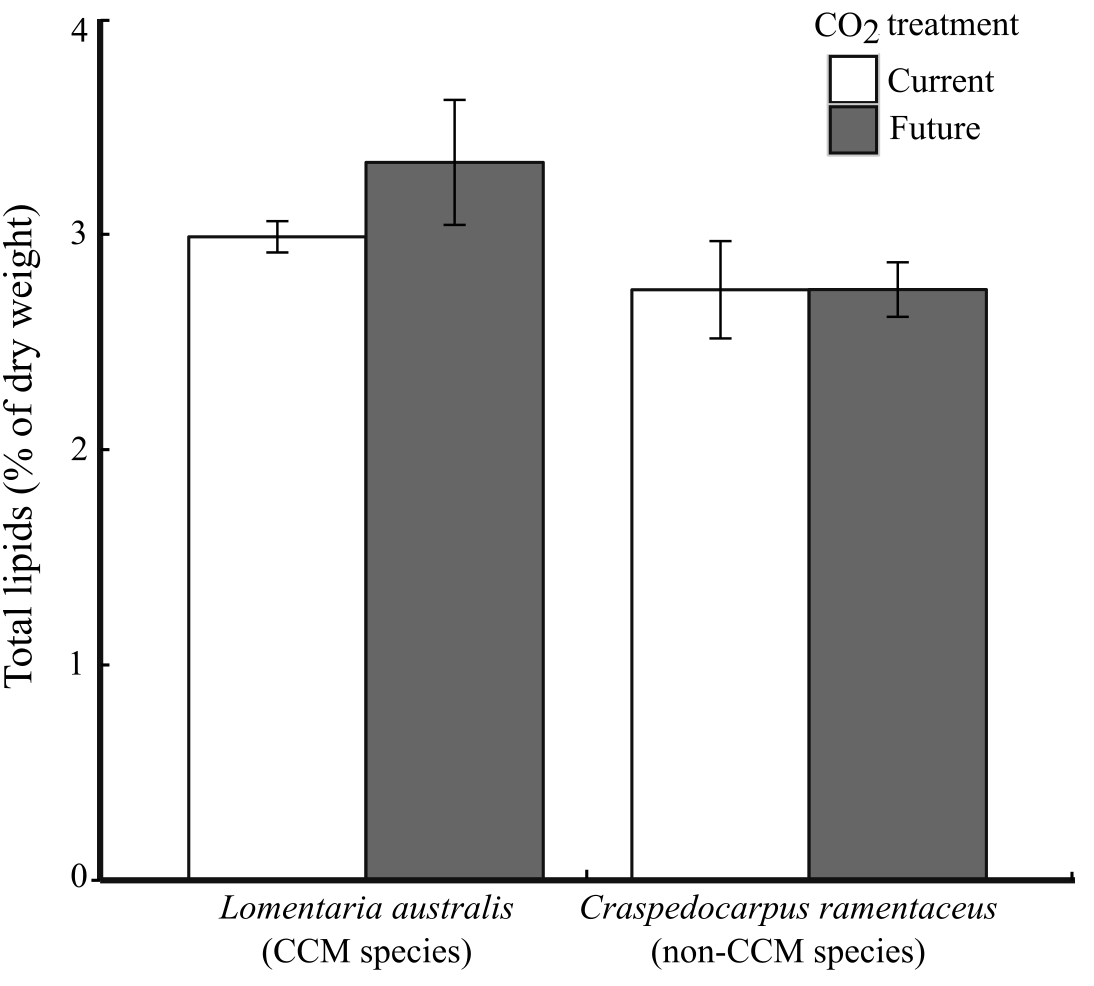


**Figure S5.** Total fatty acid content (as % of dry weight) of a species with carbon concentrating mechanism (*Lomentaria australis*; CCM species) and a species without (*Craspedocarpus ramentaceus*; non-CCM species), with current (8.0) and future (7.7) CO_2_ treatment. Data are displayed as mean ± standard error, n = 6.


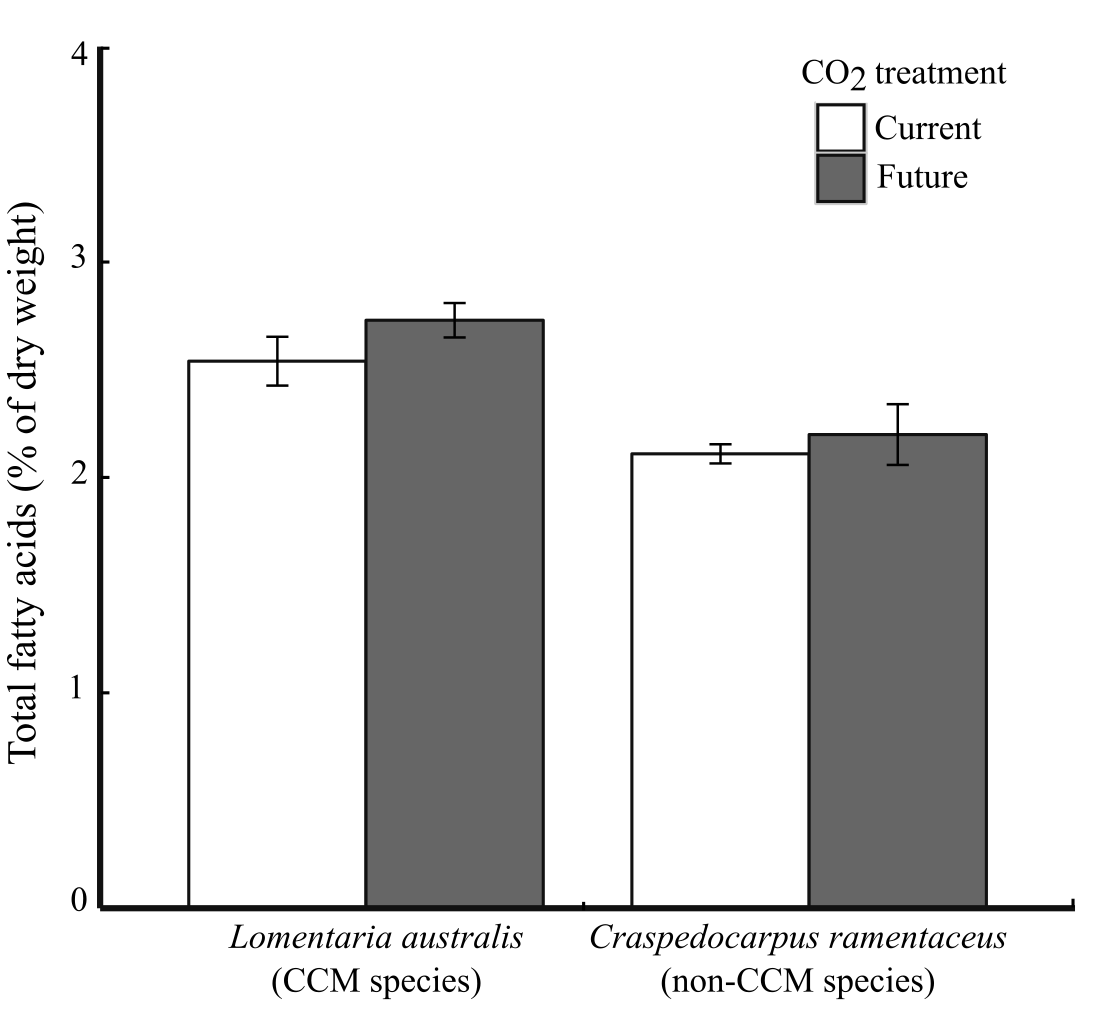


**Figure S6.** Total lipid content (as % of dry weight) of a species with carbon concentrating mechanism (*Lomentaria australis*; CCM species) and a species without (*Craspedocarpus ramentaceus*; non-CCM species), with current (8.0) and future (7.7) CO_2_ treatment. Data are displayed as mean ± standard error, n = 6.


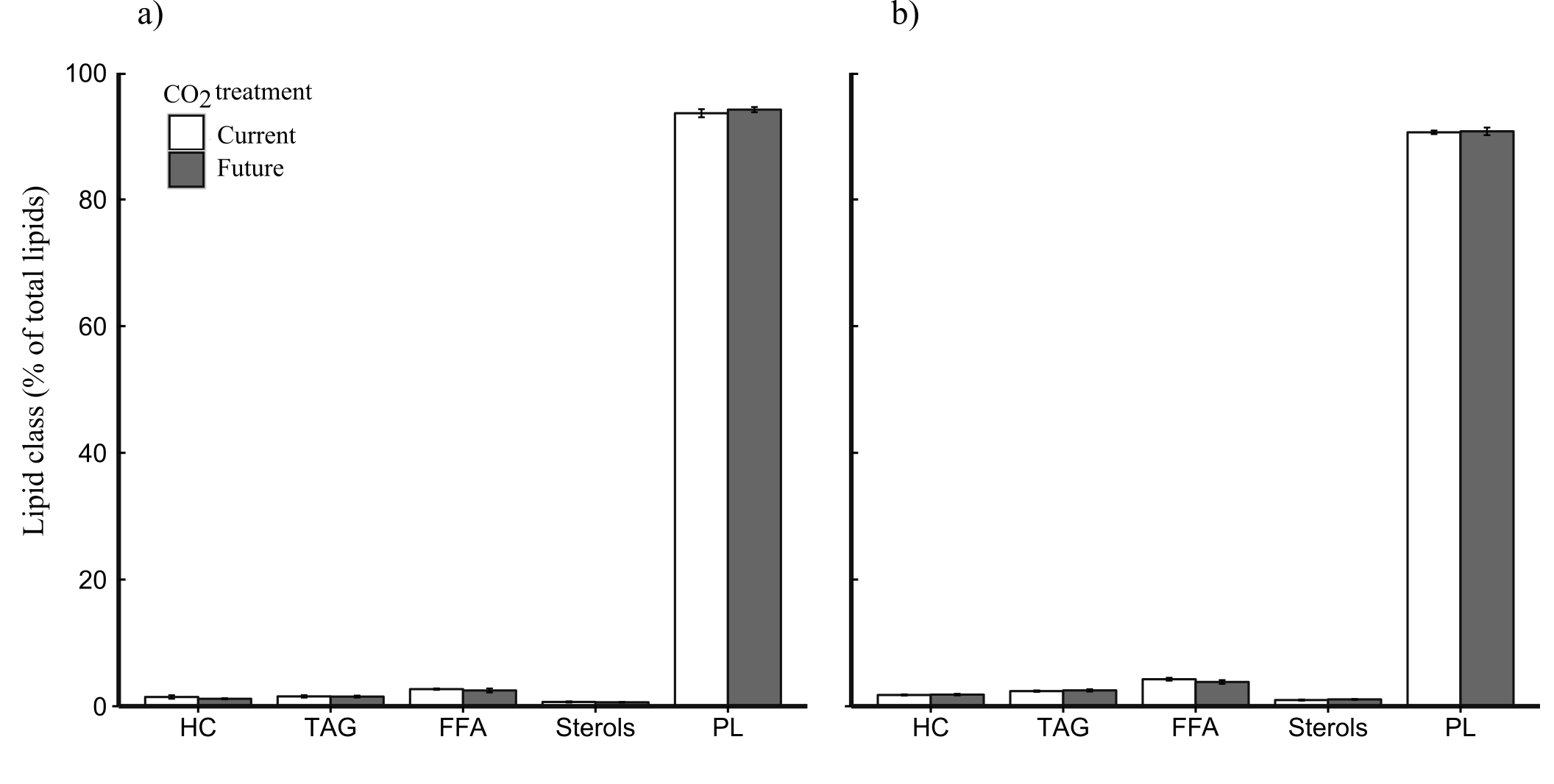


**Figure S7.** Lipid class composition (HC (hydrocarbons), TAG (triacylglycerol), Sterols, FFA (free fatty acids) and PL (polar lipids)) expressed as a percentage of total lipids, under current and future treatment, conditions with a) a species with carbon dioxide concentrating mechanism (*Lomentaria australis*; CCM species) and b) a species without carbon dioxide concentrating mechanism (*Craspedocarpus ramentaceus*; non-CCM species). Data are displayed as mean ± standard error, n = 6.

**Supplementary Tables:**

**Table S4. Stable isotopes and total tissue C and N content.** Measured on day 1 (n=23) and day 7 (n=24), for both the species with carbon dioxide concentrating mechanism (CCM) and the species without a carbon dioxide concentrating mechanism (non-CCM) under current (8.04) and future (7.70) CO_2_ treatment. Standard error in parentheses.

| **Parameters** | **CCM (Current)** | | **CCM (Future)** | | **non-CCM (Current)** | | **non-CCM (Future)** | |
| --- | --- | --- | --- | --- | --- | --- | --- | --- |
|  | **Day 1** | **Day 7** | **Day 1** | **Day 7** | **Day 1** | **Day 7** | **Day 1** | **Day 7** |
| δ^13^C (‰) | -25.47 (0.34) | -27.01 (0.19) | -23.27 (0.32) | -26.11 (0.43) | -34.37 (0.32) | -35.33 (0.18) | -34.24 (0.28) | -35.80 (0.13) |
| Carbon  (% w/w) | 23.60 (0.13) | 27.44 (0.34) | 24.25 (0.46) | 28.12 (0.43) | 23.77 (0.38) | 28.32 (0.48) | 23.77 (0.55) | 28.12 (0.22) |
| Nitrogen (% w/w) | 3.60 (0.02) | 3.21 (0.04) | 3.61 (0.09) | 3.25 (0.09) | 3.64 (0.08) | 3.43 (0.09) | 3.49 (0.06) | 3.25 (0.03) |
| C:N w/w ratio | 6.56 (0.04) | 8.55 (0.07) | 6.74 (0.12) | 8.68 (0.18) | 6.54 (0.11) | 8.29 (0.21) | 6.80 (0.11) | 8.67 (0.12) |

**Table S5. Change in carbonate chemistry parameters.** The initial value and delta (Δ) value (change over a period of 4 hours) for carbon chemistry parameters for both the species with carbon dioxide concentrating mechanism (CCM, n=48) and the species without carbon dioxide concentrating mechanism (non-CCM, n=48) under current and future conditions. Standard error in parentheses.

| **Parameters** | **Current CCM** | **Future CCM** | **Current non-CCM** | **Future non-CCM** |
| --- | --- | --- | --- | --- |
| [H^+^]_initial_, nM | 8.22 (0.08) | 21.0 (2.9) | 8.33 (0.10) | 18.31 (0.39) |
| Δ[H^+^] | -1.49 (0.13) | -5.45 (0.82) | -1.39 (0.10) | -4.48 (0.37) |
| DIC_initial_, μmol kg^-1^ | 2098.2 (3.2) | 2242 (12) | 2105.4 (2.1) | 2230.6 (5.5) |
| Δ DIC | -30.6 (3.3) | -31.3 (2.9) | -22.7 (3.2) | -33.5 (3.4) |
| HCO_3_^-^_initial_, μmol kg^-1^ | 1919.0 (3.4) | 2123 (11) | 1927.2 (2.8) | 2113.5 (6.1) |
| ΔHCO_3_^-^ | -58.4 (5.3) | -45.1 (4.4) | -48.0 (3.7) | -48.2 (4.3) |
| CO_3_^2-^_initial_, μmol kg^-1^ | 164.79 (0.15) | 78.0 (4.5) | 163.5 (1.9) | 81.8 (1.6) |
| ΔCO_3_^2-^, μmol kg^-1^ | 30.8 (3.2) | 25.0 (2.0) | 28.1 (2.3) | 24.0 (1.9) |
| CO2 _initial_, μmol kg^-1^ | 14.38 (0.15) | 40.9 (6.0) | 14.62 (0.20) | 35.27 (0.85) |
| ΔCO_2_ | -2.95 (0.25) | -11.2 (1.7) | -2.74 (0.19) | -9.24 (0.76) |

**References cited in the supplementary information:**

Cornwall, C.E., Revill, A.T., Hurd, C.L., 2015. High prevalence of diffusive uptake of CO2 by macroalgae in a temperate subtidal ecosystem. Photosynthesis Research 124, 181–190. https://doi.org/10.1007/s11120-015-0114-0

Hepburn, C.D., Pritchard, D.W., Cornwall, C.E., Mcleod, R.J., Beardall, J., Raven, J.A., Hurd, C.L., 2011. Diversity of carbon use strategies in a kelp forest community: implications for a high CO2 ocean. Global Change Biology 17, 2488–2497. https://doi.org/10.1111/j.1365-2486.2011.02411.x

Webb, W.L., Newton, M., Starr, D., 1974. Carbon dioxide exchange of Alnus rubra: a mathematical model. Oecologia 17, 281–291. https://doi.org/10.1007/BF00345747
